# SUPPORT Tools for evidence-informed health Policymaking (STP) 3: Setting priorities for supporting evidence-informed policymaking

**DOI:** 10.1186/1478-4505-7-S1-S3

**Published:** 2009-12-16

**Authors:** John N Lavis, Andrew D Oxman, Simon Lewin, Atle Fretheim

**Affiliations:** 1 Centre for Health Economics and Policy Analysis, Department of Clinical Epidemiology and Biostatistics, and Department of Political Science, McMaster University, 1200 Main St. West, HSC-2D3, Hamilton, ON, Canada, L8N 3Z5; 2 Norwegian Knowledge Centre for the Health Services, P.O. Box 7004, St. Olavs plass, N-0130 Oslo, Norway; 3 Norwegian Knowledge Centre for the Health Services, P.O. Box 7004, St. Olavs plass, N-0130 Oslo, Norway; Health Systems Research Unit, Medical Research Council of South Africa; 4 Norwegian Knowledge Centre for the Health Services, P.O. Box 7004, St. Olavs plass, N-0130 Oslo, Norway; Section for International Health, Institute of General Practice and Community Medicine, Faculty of Medicine, University of Oslo, Norway

## Abstract

This article is part of a series written for people responsible for making decisions about health policies and programmes and for those who support these decision makers.

Policymakers have limited resources for developing – or supporting the development of – evidence-informed policies and programmes. These required resources include staff time, staff infrastructural needs (such as access to a librarian or journal article purchasing), and ongoing professional development. They may therefore prefer instead to contract out such work to independent units with more suitably skilled staff and appropriate infrastructure. However, policymakers may only have limited financial resources to do so. Regardless of whether the support for evidence-informed policymaking is provided in-house or contracted out, or whether it is centralised or decentralised, resources always need to be used wisely in order to maximise their impact. Examples of undesirable practices in a priority-setting approach include timelines to support evidence-informed policymaking being negotiated on a case-by-case basis (instead of having clear norms about the level of support that can be provided for each timeline), implicit (rather than explicit) criteria for setting priorities, ad hoc (rather than systematic and explicit) priority-setting process, and the absence of both a communications plan and a monitoring and evaluation plan. In this article, we suggest questions that can guide those setting priorities for finding and using research evidence to support evidence-informed policymaking. These are: 1. Does the approach to prioritisation make clear the timelines that have been set for addressing high-priority issues in different ways? 2. Does the approach incorporate explicit criteria for determining priorities? 3. Does the approach incorporate an explicit process for determining priorities? 4. Does the approach incorporate a communications strategy and a monitoring and evaluation plan?

## About STP

*This article is part of a series written for people responsible for making decisions about health policies and programmes and for those who support these decision makers.The series is intended to help such people to ensure that their decisions are well-informed by the best available research evidence. The SUPPORT tools and the ways in which they can be used are described in more detail in the Introduction to this series *[1]. * A glossary for the entire series is attached to each article (see Additional File *1). *Links to Spanish, Portuguese, French and Chinese translations of this series can be found on the SUPPORT website (http://www.support-collaboration.org). Feedback about how to improve this tool and others in this series is welcome, and should be sent to: STP@nokc.no.*

## Scenarios

Scenario 1: You are a senior civil servant and will be submitting a plan to the Minister about how to allocate staff and other resources in order to ensure that existing programmes are well administered, emerging issues are responded to appropriately, and that evidence-informed policymaking is well supported on high-priority issues. In the past, you have found that programme administration and reactive issue management have crowded out proactive efforts to support evidence-informed policymaking. In the plan, you want to include an approach to priority setting that will support evidence-informed policymaking.

Scenario 2: You work in the Ministry of Health and are preparing a brief report about how the Ministry’s decision support unit will serve other Ministry staff. This support ranges from providing fast-turnaround requests for the best available synthesised evidence about particular issues, through to more comprehensive evidence-informed problem assessments, options to address problems, and implementation considerations that may take several weeks or months. The report will consider how the unit will prioritise which issues will get particular types of support.

*Scenario 3: You work in an independent unit that supports the Ministry of Health in its use of evidence in policymaking. You are preparing a detailed proposal for the Ministry of Health about how the unit will prioritise those issues requiring policy briefs and policy dialogues to support evidence-informed policymaking (both these issues are the focus of the SUPPORT tools discussed in Articles 13 *[2]* and 14 *[3]).

## Background

Policymakers and stakeholders have limited resources available for developing – or supporting the development of – evidence-informed policies and programmes. Such resource constraints include staff time but there are also constraints in terms of the capacity of those who are able to support policymakers. This means that only a limited amount of skilled-staff time can be allocated to finding and using research evidence to clarify a problem, frame options to address a problem, and address how an option will be implemented (these issues are the focus of the SUPPORT tools discussed in Articles 4-6 in this series [4-6]), or to other efforts to support evidence-informed policymaking. The bulk of skilled staff time needs to be allocated to administering existing programmes and to responding to emerging issues in other ways. Resource limitations may also extend to staff infrastructural needs (such as access to a librarian or journal article purchasing), and to their continuing professional development.

**Figure 1:**
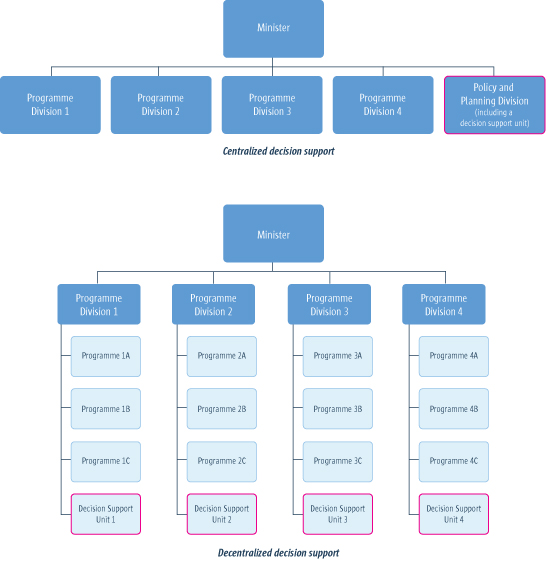
Centralized and decentralized approaches to supporting evidence-informed policymaking

Working within such resource constraints, policymakers and other stakeholders may choose to group together all staff who support evidence-informed policymaking, or else to spread them out within programme areas. Figure 1 provides a visual depiction of both a centralised approach and a decentralised approach to supporting evidence-informed policymaking. A centralised approach can facilitate the development of a common approach to priority-setting and common procedures, but it requires strong linkages with programme staff who know the issues and context well (this can be achieved potentially through the use of time-limited steering groups to oversee particular assessments of the available research evidence). A decentralised approach can facilitate the development of a culture of evidence-informed policymaking within each programme, but will require similarly strong linkages between the decision-support staff who perform similar functions in other programmes.

Policymakers and stakeholders may also choose to contract out some or all of the work to independent units with skilled staff and appropriate infrastructure. But such options also may be limited by the financial resources available. As with a centralised ‘in-house’ approach, external contracts require strong linkages with policymakers and stakeholders who know the issues and context, using possible mechanisms such as time-limited steering groups.

Whether support for evidence-informed policymaking is provided in-house or contracted out to independent units, or whether the support is centralised or decentralised, resources always need to be used wisely in order to maximise their impact. Only a very limited number of issues can be subjected to a comprehensive assessment of the available research evidence. It is important to note, too, that resource limitations also come into play when deciding which policy or programme option to pursue, or which implementation strategy to pursue (these issues are the focus of Articles 5 [5] and 6 [6] in this series). In this article, the focus is on using resources wisely to find and use research evidence to support evidence-informed policymaking.

**Figure 2:**
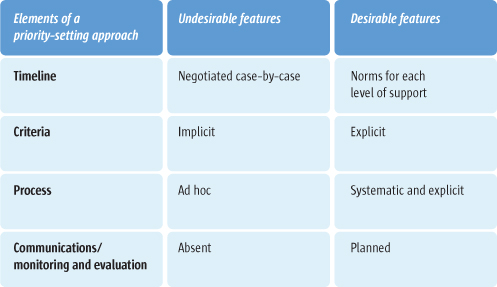
Undesirable and desirable features of a priority-setting approach

In Figure 2, the second column shows examples of possible undesirable practices which may be used in a priority-setting approach. For example, if timelines to support evidence-informed policymaking are negotiated on a case-by-case basis, policymakers will be unable to match the time constraints they face (e.g. a half-day, five-day or two-month period) to the support they could receive (a targeted search for a systematic review or a comprehensive assessment of the available research evidence). When implicit criteria are used to set priorities or the priority-setting process is ad hoc, those policymakers whose needs for research evidence are not being met may become demoralised by the lack of attention to their programme or disillusioned with the rhetoric of evidence-informed policymaking. And without either a communications plan or a monitoring and evaluation plan, policymakers will not know *why* their evidence needs are or aren’t being met, and be unable to learn whether and how the their existing approaches can be improved.

Policymakers and stakeholders charged with developing a priority-setting approach to support evidence-informed policymaking, face difficult challenges.

• They have to combine a *proactive* approach to priority setting (e.g. what priority should an issue be given in a national strategic plan for the health sector?) together with a *reactive* approach that can respond to the pressing issues of the day (e.g. what priority should an issue receive when it appears on the front page of a newspaper or is discussed in the legislature?). A priority-setting approach needs to contribute to future plans while responding to existing potentially difficult circumstances

• Policymakers have to balance *a disease or illness orientation* (e.g. what priority should be given to HIV/AIDS or diabetes?), *a programme, service and drug orientation* (e.g. what priority should be given to a screening programme, a counselling service or a new class of drugs?), and *a health system arrangements orientation* (e.g. what priority should be given to a regulatory change in the scope of the practice of nurses, or to a change in the financial arrangements that determine how doctors are paid, or to a change in the delivery arrangements that determine whether some forms of care are provided only in high-volume facilities?). A priority-setting approach needs to function with multiple, often interacting, orientations at the same time

• They have to balance shorter-term confidentiality issues with longer-term commitments to transparency and public accountability. This is particularly true for policymakers who typically rely heavily on civil servants to assess issues for them. Strict confidentiality provisions are often set to ensure that issues are not discussed before they have been vetted by policymakers. This is important given that policymakers are accountable in a very public way (through periodic elections) for the decisions they make. A priority-setting approach – at least one based within government – needs to accommodate a mix of confidentiality and transparency provisions

Some desirable practices used in a priority-setting approach for evidence-informed policymaking are derived from available tools and resources used to support priority setting in other domains. These tools and resources can be divided into three key types:

• Many tools and resources address how to prioritise illnesses and injuries. These tend to focus on the use of available data on illness and injury prevalence or incidence [7-10]

• Most tools and resources focus on how to prioritise programmes, services and drugs that are targeted at illnesses and injuries, or at ill health more generally. Many of these tools and resources focus both on data on prevalence or incidence, and on research evidence about the effectiveness or cost-effectiveness of prevention and treatment options [11-13]. Few deal with a broader set of criteria or have a more holistic approach to setting priorities [14-16]

• Almost no tools and resources address the issue of how to prioritise health system arrangements (or changes to health system arrangements) that support the provision of cost-effective programmes, services and drugs, [17] or how to prioritise actions to address the social determinants of health

Tools and resources are also available to support priority setting for both primary research and systematic reviews in the research sector [18-22] as well as for recommendations for the health sector (e.g. clinical practice guidelines) [23].

Elements of the tools and resources discussed above can be used to help to shape an approach to priority setting for those issues that will be the focus of evidence-informed policymaking. For example, burden-of-disease data may be used to inform assessments of the contribution of a particular disease to the overall burden of ill health. Research evidence about the effectiveness of programmes, services and drugs needs, can help to inform assessments of options to address ill health. Similarly, approaches to priority setting for basic research (which may use a 5-25 year time horizon), applied primary research (which may use a 2-5 year time horizon), and for systematic reviews (which may use a 6-18 month time horizon) can all provide insights into priority setting for policy briefs that are produced within a 1-6 month time horizon. (Article 13 of this series addresses the preparation and use of policy briefs in further detail) [2]. Approaches to priority setting for recommendations can also give insights into priorities for finding and using research evidence to support evidence-informed policymaking. However, a recent review of priority setting for recommendations concluded that there was “little empirical evidence to guide the choice of criteria and processes for establishing priorities” [23].

Table 1 provides examples of organisations in which a priority-setting approach can be beneficial.

**Table 1:** Examples of organisations in which an approach to setting priorities for evidence-informed policymaking can be beneficial

A number of different types of organisations have emerged to support evidence-informed policymaking. For example: • The Strategic Policy Unit, based within the United Kingdom’s Department of Health, was set up to examine high-priority issues that need to be addressed within a timeline of weeks to months • The Canadian Agency for Drugs and Therapeutics in Healthcare (http://www.cadth.ca), a national government-funded agency, provides a rapid-response function (called the Health Technology Inquiry Service) to Provincial Ministries of Health seeking input about which health technologies to introduce, cover or fund. Timelines range from 1-30 days • An Evidence-Informed Policy Network (http://www.evipnet.org) in Vietnam has obtained funding to produce two policy briefs and convene two policy dialogues in the coming year to respond to the priorities of policymakers and stakeholders • The European Observatory on Health Systems and Policies (http://www.euro.who.int/observatory) convenes a range of policy dialogues, including ‘rapid reaction seminars’ which can be organised at very short notice.http:// The On-call Facility for International Healthcare Comparisons (http://www.lshtm.ac.uk/ihc/index.html), located within the London School of Hygiene and Tropical Medicine, responds to direct requests from the United Kingdom’s Department of Health about how health systems in other high-income countries are addressing particular issues [29] Each of these organisations must, implicitly or explicitly, have timelines within which they are prepared to work. They also need criteria to decide which issues warrant significant periods of their time and which issues warrant less, or even none at all. Processes to make these decisions are also required.

## Questions to consider

The following questions can guide how to set priorities for finding and using research evidence to support evidence-informed policymaking:

1. Does the approach to prioritisation make clear the timelines that have been set for addressing high-priority issues in different ways?

2. Does the approach incorporate explicit criteria for determining priorities?

3. Does the approach incorporate an explicit process for determining priorities?

4. Does the approach incorporate a communications strategy and a monitoring and evaluation plan?

### 1. Does the approach to prioritisation make clear the timelines that have been set for addressing high-priority issues in different ways?

Policymaking processes may play out over days, weeks, or even years. Systematic and explicit priority-setting processes aren’t typically appropriate for very short timelines (i.e. hours and days) because the priority-setting process could take longer than the time in which a decision needs to be made. However, explicit criteria can still help to inform judgements about which issues require an all-hands-on-deck approach to finding and using research evidence (e.g. for those moments when a Minister says “We need it now!”). Conversely, they also help to identify which issues could be dealt with over a longer time period or should be put aside entirely, and determining which issues fall somewhere in-between.

For policymaking processes that play out over weeks or months, explicit priority setting criteria and systematic and explicit priority-setting processes can offer value. This is particularly true if there is receptivity on the part of policymakers and stakeholders to seeking an independent assessment of the research evidence (such as a policy brief) (see Article 13 for further discussion of preparing and using policy briefs to support evidence-informed policymaking) or to seeking the evidence-informed input of stakeholders through a policy dialogue (Article 14 in this series discusses how to organise and use dialogues to support evidence-informed policymaking) [2,3]. Such a priority-setting process would need to be dynamic and have revisions done every few weeks or months, if it is to provide a meaningful balance of proactive and reactive approaches.

For ‘perennial’ policy issues, and those policymaking processes that play out over many months or even years, policymakers and other stakeholders can embrace a more strategic approach to priority setting. This could include commissioning researchers to conduct a systematic review of the research literature on a specific policy or programme question, or conducting an impact evaluation of a policy or programme (this topic is the focus of Article 18 in this series) [24].

An approach to prioritisation would ideally make clear the timelines that have been set for addressing high-priority issues in different ways. Policymakers and stakeholders could then match the time constraint that they’re working under (a half-day, five-day or two-month period) to the kind of support they could receive, such as:

• A search for systematic reviews that address an issue

• A summary of the take-home messages from quality-appraised systematic reviews addressing many facets of an issue, or

• A comprehensive assessment of the research evidence available that will clarify a problem, frame options to address it, and address how an option will be implemented (i.e. a policy brief, as described in Article 13 [2])

The final column of Figure 2 highlights desirable practices that can be applied in a priority-setting approach, including the use of norms about timelines for different types of support. The other practices highlighted in this figure form the focus of Questions 2-4 below. Table 2 provides an example of timelines for (and capacity to provide) different types of support, as well as applications of the insights from questions 2-4, to the priority-setting approach used in a Ministry of Health.

**Table 2:** Example of a priority-setting approach

A Ministry’s decision-support unit offers the following range of supports to other Ministry staff: 1. A search for systematic reviews that address an issue (Timeline: 1 day; Number that can be provided per quarter: 24) 2. A summary of the take-home messages from quality-appraised systematic reviews addressing many facets of an issue (Timeline: 1 week; Number that can be provided per quarter: 12), and 3. A comprehensive assessment of the research evidence available to clarify a problem, frame options for addressing it, and address how an option will be implemented (Timeline: 1 month; Number that can be provided per quarter: 3) The unit maintains an inventory of requests, in which each request is allocated a score of between 0 and 56. On receipt, a request is reviewed by two unit staff who assign it a rating of between 1 and 7 points (where a rating of 1 indicates ‘strongly disagree’ and 7 is ‘strongly agree’) for each of the following three criteria: • The underlying problem(s), if properly addressed, could lead to health benefits, improvements in health equity or other positive impacts now or in the future, • Viable options, if properly implemented, could affect the underlying problem(s), and hence lead to health benefits, improvements in health equity or other positive impacts, or could lead to reductions in harms, cost savings or increased value for money, and • Political events could open (or political events may already have opened) windows of opportunity for change The individual scores for the third criterion are doubled, as this is deemed to be twice as important as the other two (as a way of ensuring that the Minister’s priorities are given adequate consideration). A maximum of 14 points can be assigned to criterion 1, 14 points to criterion 2, and 28 point to criterion 3. One of the two unit staff will note the nature of the support requested (support types 1, 2 or 3 above). The basis for these assessments is the request description and justification submitted by other Ministry staff (after approval from their respective divisional director). The request must address each of the three criteria using available data and evidence (when available) and a discussion about the application of explicit criteria to the issues that are considered for prioritisation At the beginning of each week, the unit manager, together with all divisional directors, reviews the rank-ordered list of priorities for each of support types 1, 2 and 3. Collectively, they confirm that the top two requests for support type 1 will proceed that week and that the top request for support type 2 will proceed. They also confirm that the top request for support type 3 is on track and that preparations are being made to begin a new assessment for the second-ranked request type 3 as soon as the current assessment is completed. The unit manager (who has training in health policy research) facilitates the meeting, taking care to elicit the rationale for any ranking changes and to ensure that any requests for comprehensive assessments are well thought through in terms of the provisional problem clarification, options framing, and implementation considerations. The unit manager then posts the decisions and rankings on the Ministry’s intranet and directs Ministry staff whose requests have not been addressed within one month of submission to submit an updated request. Once a month, the unit manager reviews the unit’s monitoring data with the divisional directors. The monitoring data includes the number of appeals submitted by Ministry staff and their resolution. Once every year, the unit re-evaluates the scale of its outputs to determine if it can provide more support within shorter time frames. Once every three years, the unit commissions an evaluation of its impacts on the policymaking process.

### 2. Does the process incorporate explicit criteria for determining priorities?

Explicit criteria can help to guide those involved in a priority-setting process *and*, if confidentiality restrictions permit, in communicating the rationale for decisions about priorities to other policymakers and stakeholders. Three possible criteria for prioritising a given issue include:

• The underlying problem(s), if properly addressed, could lead to health benefits, improvements in health equity or other positive impacts, now or in the future

• Viable options, if properly implemented, could affect the underlying problem(s), and hence lead to health benefits, improvements in health equity or other positive impacts, or could lead to reductions in harms, cost savings or increased value for money, and

• Political events could open (or political events may already have opened) ‘windows of opportunity’ for change. For example, in 1993 Taiwan’s President submitted a national health insurance bill to Parliament in order to pre-empt a challenge by an opposition party [25]. The pending challenge opened a significant window of opportunity for change, and for finding and using research evidence to support policymaking about national health insurance

The application of these criteria requires readily available data and research evidence, as well as collective judgement (based on these and other considerations) about whether an issue warrants prioritisation. A thorough assessment would only be needed for a limited range of issues considered to be of higher priority.

The first criterion listed above relates, in part, to concerns such as the burden of illness and the likely severity of new or emerging illnesses. But it also relates to judgements about how likely it is that the underlying problem(s) can be addressed. These underlying problem(s) may vary in scope, ranging from a narrow focus on the specific characteristics of particular illnesses and injuries, through to the programmes, services and drugs used to prevent or treat these illnesses and injuries, and/or the health system arrangements that support the provision of programmes, services or drugs. Given that data and research evidence about underlying problem(s) may not be readily available or may be lacking entirely, other considerations may need to be introduced. (Article 4 in this series provides an overview of the processes involved in using research evidence to clarify problems) [4].

The second criterion requires judgement about how likely it is that options will have acceptable costs and desired consequences (i.e. how likely it is that they would be considered viable). Framing options to address a problem – the focus of Article 5 in this series – requires systematic reviews of studies to examine the benefits and harms of options, as well as data or research evidence about costs and cost-effectiveness [5]. Two recent developments, namely the growth of databases containing systematic reviews and the growing availability of policymaker-friendly summaries of systematic reviews that can be linked to from these databases (which are the focus of Article 7), have made preliminary assessments of this type increasingly feasible [26]. However, where research evidence about the viability of options is not readily available, other considerations will need to be introduced.

The third criterion requires judgement about whether a window of opportunity for action could open, or has opened [27]. As we review further in Article 4, such opportunities can occur because of the attention that is given to a problem at particular moments in time [4]. Significant media coverage, for example, may be given to documented cases of significant gaps in quality and access in cancer care delivery. These windows, however, can close equally fast because media attention tends to move on quickly. Windows of opportunity may also be opened by political events, such as, for example, the formation of a coalition of stakeholders who have chosen to take action on a particular issue, or when a politician with a personal interest in an issue is appointed as a Minister of Health. Some events related to problems or politics can be predicted, such as the publication of periodic reports by national statistical agencies, the development of a national health sector strategic plan, and the setting of annual budgets, as well as elections. But often the specific *nature* of the opportunity can’t be.

### 3. Does the process incorporate an explicit process for determining priorities?

Explicit criteria do not make decisions – people do. And a systematic and explicit process can help them to make decisions in a defensible way. Four possible desirable features of a priority-setting process include:

• It is informed by a pre-circulated summary of available data and evidence and by a discussion about the application of explicit criteria to issues that are considered for prioritisation

• It ensures fair representation of those involved in, or affected by, future decisions about the issues that are considered for prioritisation

• A facilitator is engaged who uses well-constructed questions to elicit views about the priority that should be accorded to issues as well as the rationale for their prioritisation, and

• An experienced team of policymakers and researchers is engaged to turn high-priority issues into clearly defined problem(s) and viable options that will be the focus of more detailed assessments

The preparation of a pre-circulated summary of available data and evidence about possible priority issues is a highly efficient way of preparing participants for a priority-setting process. Gaps in the data and research evidence can be as important to describe as what is available. Such summaries can provide common ground for discussions.

A priority-setting process would ideally bring together the many parties involved in, or affected by, any future decisions related to the issues that are under consideration as possible priorities. Doing this requires careful mapping of the full range of stakeholders and then selecting appropriate individuals from different stakeholder groups of. Confidentiality provisions may be particularly challenging in this process if they preclude the involvement of those who will be affected by any future decisions related to the issues concerned. Civil servants, and especially politicians, may then be required to participate on their behalf.

A skilled, knowledgeable and neutral facilitator is required to ensure that a priority-setting process runs well. In Article 14 in this series, we describe the rationale for this combination of attributes [3]. For a priority-setting process that is entirely internal to government, it may be ideal if the facilitator is drawn from a decision-support unit, rather than from divisions in charge of particular policy domains (e.g. human resources policy) or particular programmes (e.g. diabetes care).

An experienced team of policymakers and researchers is required to turn high-priority issues into clearly defined problem(s) as well as viable options that will form the focus of more detailed assessments. The team would ideally establish clear timelines for each issue that needs to be addressed. The team could also provide guidance about which issues could be addressed in-house, and which could be contracted out. If certain issues are deemed confidential, these too could either be dealt with in-house or contracted out with clearly stated confidentiality clauses in the work contracts.

While this process may sound complex, as described in Table 2, it can be operationalised in a very practical way in a given setting.

### 4. Does the process incorporate a communications strategy and a monitoring and evaluation plan?

A communications strategy is needed to ensure that policymakers and stakeholders are informed of the high-priority issues so that they can prepare input into the further clarification of the problems, the framing of options, and addressing how an option will be implemented. Ideally, a range of materials, fine-tuned for different stakeholders, would be produced as part of the communications strategy. However, in some contexts or for some issues, confidentiality provisions may not permit communication with certain stakeholders.

Even the best communications strategy will not reach everyone and it may not elicit the desired commitment to address the high-priority issues. A monitoring plan can help to address this by identifying when high-priority issues are not being addressed within the established timeframe. An accompanying evaluation plan can be used to examine particular issues in a more systematic way, such as the impacts of the priority-setting process on the policymaking process, and how and why stakeholders respond to the priorities identified.

## Conclusion

Setting priorities for finding and using research evidence to support evidence-informed policymaking can all too easily be skipped over entirely or done too rapidly or in too cursory a way. Moreover, the selected approach to priority setting may not be implemented or it may not be implemented fully. It may also not possible to repeat a particular approach periodically given that windows of opportunity may open and close at different times. Any such failures in priority setting may mean that significant opportunities to support evidence-informed policymaking are missed and that the culture of evidence-informed policymaking is eroded. Close attention should therefore be paid to whether timelines for addressing high-priority issues in different ways are realistic and are being met, whether the criteria and process chosen for determining priorities are realistic and being used, and whether a communications strategy and monitoring and evaluation plan have been developed and are being implemented. Even in highly resource-constrained environments, attention to such issues is likely to ensure that existing resources to support evidence-informed policymaking are directed to where they can have the biggest impact.

## Resources

### Useful documents and further reading

- Healy J, Maxwell J, Hong PK, Lin V: *Responding to Requests for Information on Health Systems from Policy Makers in Asian Countries*. Geneva, Switzerland: Alliance for Health Policy and Systems Research, World Health Organization; 2007 [28]. – Source of lessons learned about organisations that support evidence-informed policymaking, but with little attention given to how priorities are set by these organisations (http://www.who.int/alliance-hpsr/RespondingRequests_HS_AsianCountries_Healy.pdf)

- Nolte E, Ettelt S, Thomson S, Mays N: Learning from other countries: An on-call facility for health care policy. *Journal of Health Services Research and Policy* 2008, 13 (supp 2): 58-64 [29]. – Source of lessons learned by an independent organisation that supports evidence-informed policymaking, with some attention given to how priorities are set by the organisation

### Links to websites

- Global burden of disease: http://www.who.int/topics/global_burden_of_disease/en – Source of data and research evidence about the global burden of disease. This information can be one input among many in priority setting for evidence-informed policymaking.

- Disease Control Priorities Project: http://www.dcp2.org/main/Home.html – Source of research evidence and recommendations about the programmes, services and drugs that should be prioritised in different types of countries. This information can be one input among many in priority setting for evidence-informed policymaking.

- CHOosing Interventions that are Cost-Effective (CHOICE): http://www.who.int/choice/en – Source of data, research evidence and a tool about the programmes, services and drugs that should be prioritised in different regions and countries. This information can be one input among many in priority setting for evidence-informed policymaking.

- Canadian Priority Setting Research Network: http://www.canadianprioritysetting.ca – Source of published articles about priority-setting in healthcare, which may provide lessons for priority setting for evidence-informed policymaking.

## Competing interests

The authors declare that they have no competing interests.

## Authors’ contributions

JNL prepared the first draft of this article. ADO, SL and AF contributed to drafting and revising it.

## Supplementary Material

Additional File Glossary Click here for file
